# 5XFAD Mice Show Early Onset Gap Detection Deficits

**DOI:** 10.3389/fnagi.2019.00066

**Published:** 2019-04-02

**Authors:** Katherine Kaylegian, Amanda J. Stebritz, Aldis P. Weible, Michael Wehr

**Affiliations:** Department of Psychology, Institute of Neuroscience, University of Oregon, Eugene, OR, United States

**Keywords:** Alzheimer's, gap detection, mouse model, auditory processing, acoustic startle response

## Abstract

Alzheimer's patients show auditory temporal processing deficits very early in disease progression, before the onset of major cognitive impairments. In addition to potentially contributing to speech perception and communication deficits in patients, this also represents a potential early biomarker for Alzheimer's. For this reason, tests of temporal processing such as gap detection have been proposed as an early diagnosis tool. For a biomarker such as gap detection deficits to have maximum clinical value, it is important to understand what underlying neuropathology it reflects. For example, temporal processing deficits could arise from alterations at cortical, midbrain, or brainstem levels. Mouse models of Alzheimer's disease can provide the ability to reveal in detail the molecular and circuit pathology underlying disease symptoms. Here we tested whether 5XFAD mice, a leading Alzheimer's mouse model, exhibit impaired temporal processing. We found that 5XFAD mice showed robust gap detection deficits. Gap detection deficits were first detectable at about 2 months of age and became progressively worse, especially for males and for longer gap durations. We conclude that 5XFAD mice are well-suited to serve as a model for understanding the circuit mechanisms that contribute to Alzheimer's-related gap detection deficits.

## Introduction

A major challenge faced by Alzheimer's patients is a decline in communication skills. Recent findings indicate that these deficits are not simply due to general cognitive impairment. Rather, there appear to be specific impairments of auditory perception which precede full-blown Alzheimer's disease (Gates et al., 2002; Swords et al., 2018). One such deficit shown by adults with Mild Cognitive Impairment (MCI) is a deficit in detecting gaps in background noise (Iliadou et al., 2017). Gap detection is a measure of temporal acuity that is correlated with speech perception deficits in older adults (Glasberg et al., 1987; Fitzgibbons and Gordon-Salant, 1996; Snell and Frisina, 2000). Because speech segmentation and phoneme identification depend on gaps and other temporal cues in speech sounds, gap detection serves as a model for speech processing. Because patients show a gap detection deficit early, in the MCI phase, this measure has been proposed as a biomarker that could achieve earlier diagnosis of dementia (Iliadou et al., 2017). Although not all MCI patients go on to develop Alzheimer's, longitudinal studies have shown that deficits in central auditory processing (such as dichotic listening) can predict the subsequent development of Alzheimer's disease with a relative risk ratio of up to 23 (Gates et al., 2002).

The mechanisms underlying these auditory processing deficits in MCI patients remain unknown. Could gap detection deficits be a sign of early neurodegeneration? Alzheimer's disease in humans, as well as in many mouse models, is marked by amyloid plaques and neurofibrillary tangles, synapse loss, axonopathy, gliosis, and neuronal loss (Hall and Roberson, 2012). Although it seems likely that such changes could lead to auditory processing deficits, it is unclear how auditory circuits are specifically altered and what impact this has on neural computation. Both cortical and subcortical changes have been correlated with speech encoding and gap detection deficits in Alzheimer's patients (Bidelman et al., 2017; Tuwaig et al., 2017), but it remains unclear whether and how cortical and/or subcortical changes contribute to auditory processing deficits.

There is a growing consensus that prevention or treatment of dementia due to Alzheimer's disease will require interventions prior to the onset of cognitive symptoms (Jack and Holtzman, 2013; Tuwaig et al., 2017). Biomarkers such as gap detection deficits could be instrumental for early diagnosis. However, a truly valuable biomarker should go beyond correlations with disease measures, and it is therefore critical to understand which brain changes the biomarker reflects and how these are mechanistically related to Alzheimer's pathology.

Mouse models of Alzheimer's disease have helped to reveal the molecular and circuit pathology underlying disease symptoms (Hall and Roberson, 2012). Recent progress in understanding the cortical and subcortical circuit mechanisms underlying gap detection in the mouse auditory system (Walton et al., 1997; Weible et al., 2014a,b; Anderson and Linden, 2016; Keller et al., 2018) raise the possibility that this model system could provide mechanistic insight into how Alzheimer's pathology produces gap detection deficits. As a first step toward this goal, here we tested gap detection in the 5XFAD mouse model of Alzheimer's disease. These mice have five familial Alzheimer's disease mutations and develop amyloid deposition starting at about 1.5–2 months of age, and neurodegeneration and cognitive deficits as early as 4–5 months of age (Oakley et al., 2006; Hall and Roberson, 2012). We found that 5XFAD mice showed a robust gap detection deficit. Gap detection deficits first appeared at about 2 months of age and progressively deteriorated, especially for males and for longer gap durations. We conclude that 5XFAD mice are well-suited to serve as a model for understanding the circuit mechanisms that contribute to Alzheimer's-related gap detection deficits.

## Results

**Figure 1:**
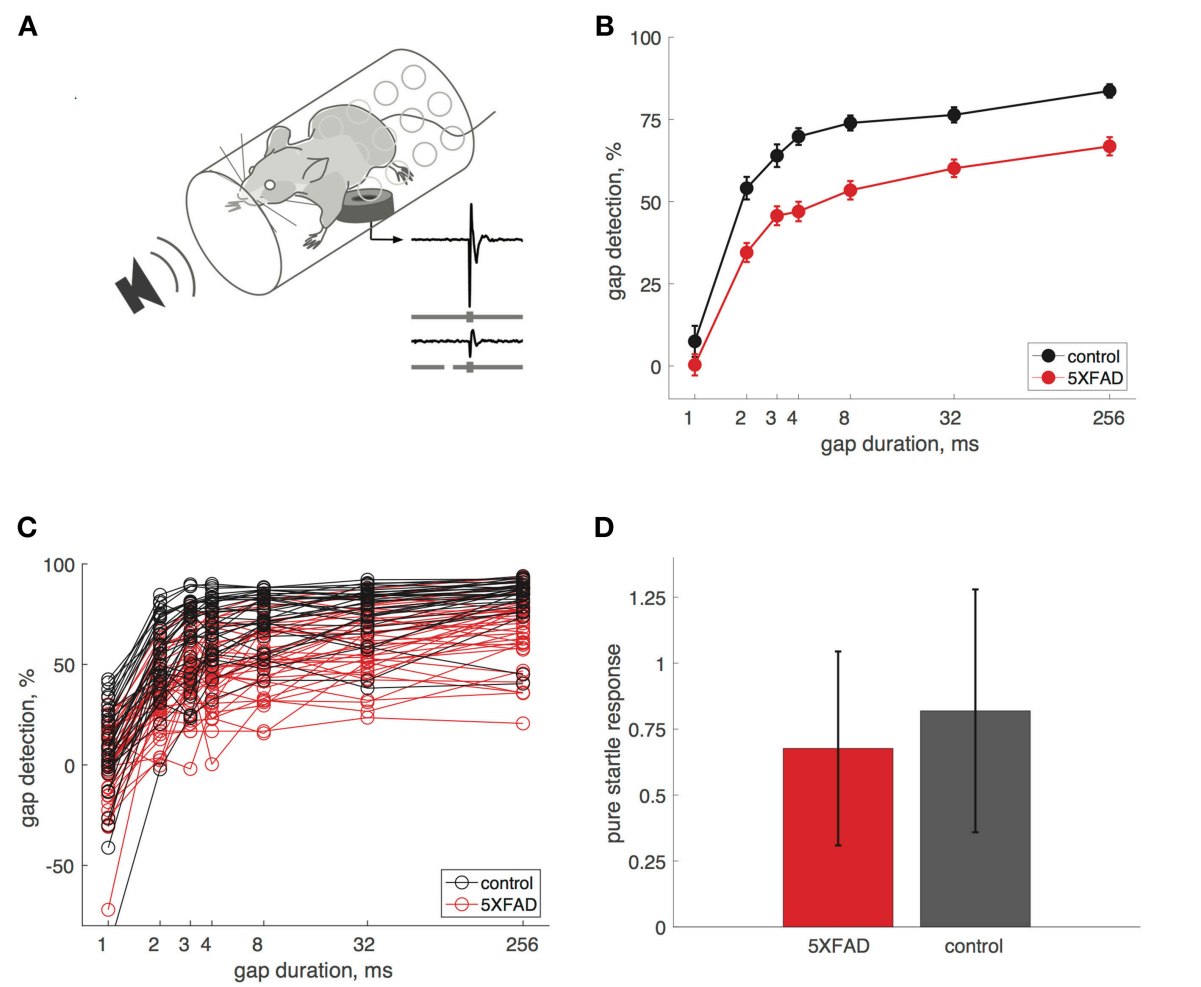
Gap detection was impaired in 5XFAD mice. **(A)** We measured startle responses by placing mice in a perforated tube resting on a pressure sensor. A gap in continuous background noise attenuates the startle response evoked by a burst of noise, presented 50 ms after the gap. We measured gap detection as the percentage reduction in the startle response compared to trials without a gap. Traces show example startle responses and gray bars depict stimuli without a gap (top) and with a 32 ms gap (bottom). **(B)** Gap detection was impaired in 5XFAD mice (red) compared to littermate controls (black). Error bars show s.e.m. across sessions. Data in **B–D** are from adult mice (age >60 days). **(C)** Gap detection for all sessions in individual mice. Note that negative gap detection values correspond to startle facilitation by the gap (e.g., for some 1 ms gap responses). 5XFAD and control mice did not differ in the occurrence of negative gap detection values (χ^2^ = 0.9, *p* = 0.34). **(D)** Pure startle responses (with no preceding gap) were not significantly different for 5XFAD mice compared to controls. Startle responses are in arbitrary units. Error bars show s.d. across sessions.

We measured gap detection in mice using a variant of pre-pulse inhibition, in which a gap in continuous background noise acts as a cue that reduces the acoustic startle response (Weible et al., 2014b). We measured startle responses by placing mice in a tube resting on a pressure sensor (Figure 1A). Gaps attenuate the startle response, and we used the percentage startle reduction as our measure of gap detection. Longer gaps produced progressively stronger gap detection (Figure 1B). We first tested adult mice (older than 60 days). Gap detection thresholds in both 5XFAD and control mice were 2 ms, which is typical for mice and humans (Glasberg et al., 1987; Weible et al., 2014b). Although minimum detection thresholds were unaffected, 5XFAD mice showed robust deficits above threshold. 5XFAD mice showed strongly impaired gap detection for all gap durations at or above threshold, compared to littermate controls (Figures 1B,C). This impaired gap detection was highly significant at the group level (*p* = 1 × 10^−15^, *n* = 38 sessions in 16 5XFAD mice, *n* = 33 sessions in 15 control mice, df = 1, χ^2^ = 64, Kruskal-Wallis). 5XFAD mice were significantly impaired for all gap durations longer than 1 ms (2–256 ms, *post-hoc* rank-sum). Pure startle responses (i.e., those not preceded by a gap) were not significantly different in 5XFAD and control mice (Figure 1D, *p* = 0.19, rank-sum), suggesting that the gap detection deficit is specific to temporal processing and not due simply to hearing loss or impaired startle reflexes.

### Sex Differences

**Figure 2:**
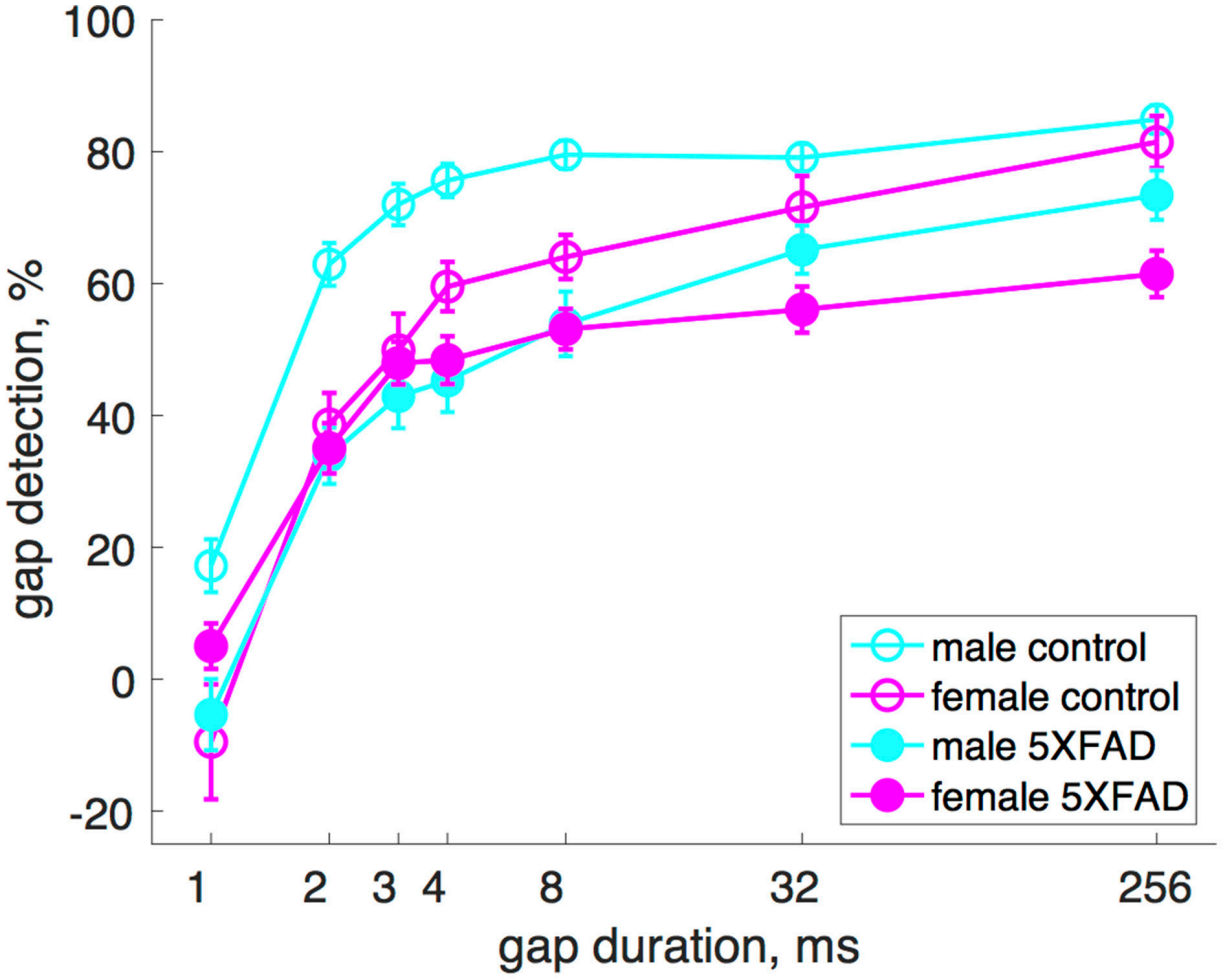
Male 5XFAD mice had more strongly impaired gap detection than 5XFAD females. Both male and female 5XFAD mice were impaired relative to male and female controls, respectively, but males were more so. Male control mice were significantly better at gap detection than female control mice. Error bars show s.e.m. across sessions.

Male 5XFAD mice were more impaired at gap detection than females (Figure 2). Across all gap durations, male 5XFAD mice showed a stronger impairment compared to male controls than did female 5XFAD mice compared to female controls (male 5XFAD vs. control: *p* = 9 × 10^−21^, *n* = 17 sessions in 8 5XFAD male mice, *n* = 21 sessions in 8 control male mice, df = 1, χ^2^ = 87; female 5XFAD vs. control: *p* = 0.004, *n* = 21 sessions in 8 5XFAD female mice, *n* = 12 sessions in 7 control female mice, df = 1, χ^2^ = 8, Kruskal-Wallis). We also noted that male control mice were significantly better at gap detection than female control mice (*p* = 6 × 10^−6^, df = 1, χ^2^ = 19.3, Kruskal-Wallis).

### Effects of Age

**Figure 3:**
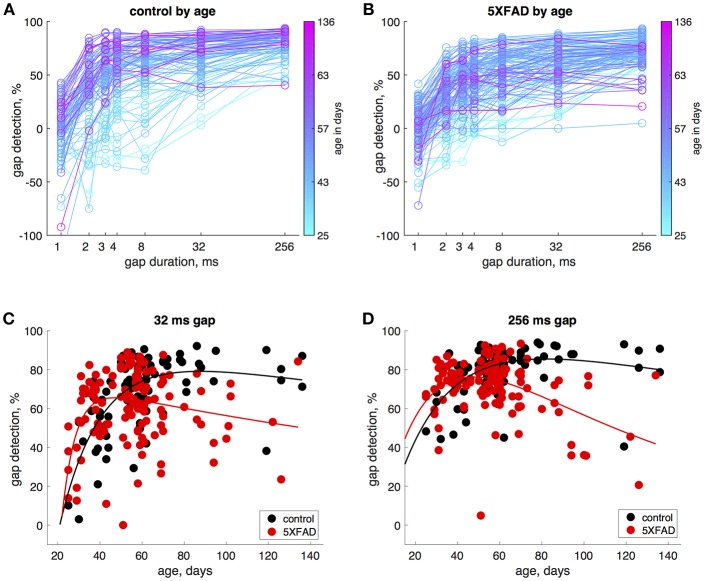
Gap detection performance developed with age in both 5XFAD and control mice, but was impaired in adulthood in 5XFAD mice. **(A)** We tested gap detection in 25 control mice from ages 25–136 days. Gap detection is shown for 91 individual sessions, color-coded by age. **(B)** Gap detection in 28 5XFAD mice over a similar age range (25–134 days, 126 sessions). **(C)** Gap detection performance as a function of age for 32 ms gaps. Solid lines show a two-exponential fit (see Methods). Both 5XFAD and control mice improved with age up to about 60 days of age, after which the deficit in 5XFAD mice became apparent. Gap durations of 2–32 ms produced very similar patterns of gap detection. **(D)** For 256 ms gaps, gap detection improved with age up 60 days of age for both 5XFAD and control, and then declined significantly for 5XFAD mice.

In control mice, gap detection improved with age. To examine this more closely, we tested mice approximately weekly from ages 25 to 136 days. Gap detection performance in control mice increased with age up until about 60 days, after which performance remained stable (Figure 3A). This was true for all gap durations above threshold (>1 ms). In 5XFAD mice, gap detection also improved with age up to about 60 days, but reached a lower steady-state performance level (Figure 3B). For the longest duration (256 ms), gap detection performance declined with age after 60 days. Figures 3C,D show gap detection performance as a function of age for two representative gap durations (32 and 256 ms). The developmental improvement up to about 60 days in both control and 5XFAD mice, followed by age-related deterioration in 5XFAD mice, were well-fit by a two-exponential model (Figures 3C,D). The deficit in 5XFAD mice compared to controls emerged as early as 60 days of age (for 256 ms gaps, *p* = 0.04, 5XFAD: *n* = 78 sessions in 20 mice, control: *n* = 54 sessions in 16 mice, rank-sum). Linear regression of gap detection as a function of age older than 60 days showed a significant decline for 256 ms gaps (*r*^2^ = 0.3, *p* = 0.0004), but not for other gap durations (Table 1). Taken together, these results show that the 5XFAD mutations cause an age-related gap detection deficit that is marked by impaired detection of short gaps by adult mice, combined with deteriorating detection of long gaps. It is possible that age-related deterioration could become evident even for shorter gaps as mice continue to age beyond 136 days (~5 months).

**Table 1:** Linear regression of gap detection as a function of age.

	**Gap duration (ms)**	**Younger than 60 days**				**Older than 60 days**			
		**Slope**	***r*^**2**^**	***p***	***n***	**Slope**	***r*^**2**^**	***p***	***n***
5XFAD	1	0.50	0.09	0.0035	88 sessions, 22 mice	−0.29	0.08	0.0811	38 sessions, 16 mice
	2	1.17	0.28	0.0000	88 sessions, 22 mice	−0.01	0.00	0.9594	38 sessions, 16 mice
	3	1.15	0.31	0.0000	88 sessions, 22 mice	−0.07	0.01	0.6354	38 sessions, 16 mice
	4	1.09	0.28	0.0000	88 sessions, 22 mice	−0.09	0.01	0.5656	38 sessions, 16 mice
	8	1.02	0.24	0.0000	88 sessions, 22 mice	−0.23	0.06	0.1239	38 sessions, 16 mice
	32	0.53	0.09	0.0041	88 sessions, 22 mice	−0.16	0.03	0.2653	38 sessions, 16 mice
	256	0.19	0.03	0.1152	88 sessions, 22 mice	−0.48	0.30	0.0004	38 sessions, 16 mice
Control	1	0.55	0.04	0.1504	58 sessions, 18 mice	−0.10	0.01	0.6466	33 sessions, 15 mice
	2	2.26	0.46	0.0000	58 sessions, 18 mice	−0.08	0.01	0.6035	33 sessions, 15 mice
	3	2.21	0.51	0.0000	58 sessions, 18 mice	0.05	0.00	0.7469	33 sessions, 15 mice
	4	2.39	0.60	0.0000	58 sessions, 18 mice	0.00	0.00	0.9669	33 sessions, 15 mice
	8	2.39	0.56	0.0000	58 sessions, 18 mice	−0.01	0.00	0.8897	33 sessions, 15 mice
	32	1.27	0.39	0.0000	58 sessions, 18 mice	0.03	0.00	0.7305	33 sessions, 15 mice
	256	0.66	0.30	0.0000	58 sessions, 18 mice	−0.02	0.00	0.8150	33 sessions, 15 mice

*We separately fit ages older or younger than 60 days. In younger mice, both 5XFAD and control mice showed significantly positive slopes for nearly all gap durations, reflecting the developmental increase in gap detection performance from 25 to 60 days. In older control mice, regression slopes were not significantly different from 0, indicating stable performance in adulthood. In older 5XFAD mice, slopes were weakly negative and not significantly different from 0, except for detection of 256 ms gaps which showed significant deterioration with age. However, regression slopes for old 5XFAD mice were significantly more negative than slopes for old controls (p = 0.013, 1-tailed rank-sum)*.

Finally, to jointly model the effects of genotype, sex, age, and gap duration on gap detection performance, we used a generalized linear mixed-effects model (Table 2). This analysis estimates the effects of each of these terms on gap detection after accounting for the random effects of differences across individual mice. The main effects of genotype, age, and gap duration were highly significant, and there were significant interactions between genotype and age, genotype and gap duration, genotype and sex, and between gap duration and age over 60 days.

**Table 2:** Generalized piecewise linear mixed effects model.

**Term**	**β**	***F***	**DF_**1**_**	**DF_**2**_**	***p***
**(Intercept)**	**−94.538**	**49.471**	**1**	**1504**	**3.0455E-12**
**genotype**	**38.581**	**18.416**	**1**	**1504**	**1.8886E-05**
**gap duration**	**13.64**	**147.02**	**1**	**1504**	**2.381E-32**
**age_early**	**1.5348**	**23.94**	**1**	**1504**	**1.1007E-06**
**age_late**	**1.2098**	**33.545**	**1**	**1504**	**8.4642E-09**
sex	4.1868	0.072318	1	1504	0.78803
**genotype:gap duration**	**−1.1528**	**5.0448**	**1**	**1504**	**0.024844**
**genotype:age_early**	**−0.46575**	**7.4728**	**1**	**1504**	**0.0063373**
gap duration:age_early	−0.038677	3.132	1	1504	0.07697
**genotype:age_late**	**−0.48945**	**12.541**	**1**	**1504**	**0.00041034**
**gap duration:age_late**	**−0.04201**	**9.9691**	**1**	**1504**	**0.0016235**
**genotype:sex**	**−12.153**	**13.193**	**1**	**1504**	**0.0002904**
gap duration:sex	0.46107	0.73302	1	1504	0.39204
age_early:sex	0.020552	0.0029173	1	1504	0.95693
age_late:sex	0.20819	0.75336	1	1504	0.38555

Significant predictors are shown in bold. We fit gap detection performance as the full model given by:

*gap detection ~ genotype + gap duration + age_early + age_late + sex*

*+ genotype:gap duration + genotype:age_early + genotype:age_late*

*+ genotype:sex + gap duration:age_early + gap duration:age_late*

*+ gap duration:sex + age_early:sex + age_late:sex*

*+ (1 + gap duration | mouse) + (1 + age_early | mouse)*

*+ (1 + age_late | mouse)*

*where “~” denotes “modeled as,” “:” denotes interaction products, “|” denotes “by,” which accounts for the random effects of individual mice modeled as random intercepts (“1”) and random slopes for the dependence on gap duration and age. We fit age with a piecewise linear function split before and after 60 days (age_early and age_late). β indicates the fitted coefficients, F and p indicate the F statistic and corresponding p-value for the significance of each fixed effect term (ANOVA), and DF_1_ and DF_2_ indicate the numerator and denominator degrees of freedom for the F statistic. This analysis shows that gap detection depended significantly on genotype, age (both early and late), and gap duration, and that there were significant interactions between genotype and age (both early and late), genotype and gap duration, genotype and sex, and between gap duration and late age (over 60 days)*.

## Discussion

Here we tested gap detection in 5XFAD mice, a widely-used mouse model of Alzheimer's disease. We found that these mice showed impaired gap detection as early as age 60 days, and that this deficit became progressively stronger over time. Adult males were more strongly affected than females. Gap detection for long gaps (256 ms) declined significantly with age, whereas detection of shorter gaps was impaired in adult mice but did not decline significantly with age. These results show that 5XFAD mice, like human Alzheimer's patients, have a temporal processing deficit which appears much earlier than the onset of memory and other cognitive deficits (Oakley et al., 2006; Iliadou et al., 2017; Swords et al., 2018). This suggests that these mice will be a valuable model system for investigating the neurodegenerative mechanisms underlying impaired gap detection in Alzheimer's disease, which will be important for validating this biomarker and understanding how it relates to Alzheimer's pathology.

It's important to note that we only tested mice up to a maximum age of 136 days (~5 months), so we don't know yet whether or how gap detection deficits will progress at later ages. Based on our finding that gap detection for long gaps declined significantly across ages 60–136 days, it seems likely that further deterioration will occur. We also note that both our 5XFAD mice and their littermate controls are on a C57BL6/SJL hybrid genetic background. 5XFAD mice on this background show a more severe phenotype than those on an isogenic C57BL6 background (Hall and Roberson, 2012). The hybrid background is unlikely to show the early-onset age-related hearing loss seen in the C57BL6 background, which is due in part to a single nucleotide substitution in the Cdh23 gene (Johnson et al., 2017), but does make it more difficult to directly compare behavioral and neurophysiological results to standard mouse backgrounds such as C57BL6. Moreover, hearing loss remains a concern in 5XFAD mice (but not their littermate controls), since these mice have been reported to show hearing loss (increased auditory brainstem response thresholds) as early as 13–14 months of age, which is at least partially explained by hair cell loss (O'Leary et al., 2017). Pre-pulse inhibition and startle responses are also affected, as early as 3–4 months of age. We found that startle responses were slightly, but not significantly reduced in 5XFAD mice compared to littermate controls. This is unlikely to affect our results, because we excluded any sessions in which mice didn't show robust pure startle responses (see Methods). The fact that we saw the most age-related deterioration for long gaps (256 ms) is consistent with subcortical neurodegeneration, since lesion and optogenetic suppression studies have demonstrated a critical role for auditory cortex in the detection of brief gaps (< 64 ms) but not long gaps (Ison et al., 1991; Kelly et al., 1996; Syka et al., 2002; Bowen et al., 2003; Threlkeld et al., 2008; Weible et al., 2014b). However, our results certainly do not rule out contribution from cortical neurodegeneration. Together these findings suggest that brainstem auditory circuits are affected in 5XFAD mice.

We opted to test gap detection in 5XFAD mice because these mice show early and aggressive amyloid pathology (Oakley et al., 2006). Our results suggest that other mouse models of Alzheimer's disease may also show gap detection deficits, although these may not be as robust nor appear as early as in 5XFAD mice. Although 5XFAD mice include five human Alzheimer's mutations, they do not show tau pathology (Hall and Roberson, 2012), which could affect the precise steps underlying the progression of auditory processing deficits. It will be straightforward to test whether other mouse models of Alzheimer's disease also show gap detection or other auditory processing deficits (Hall and Roberson, 2012). The relative strength and timing of such deficits during disease progression could help reveal the importance of different mutations and pathways in contributing to this auditory processing phenotype.

We found that male 5XFAD mice showed stronger gap detection deficits than female 5XFAD mice, which was due in part to better gap detection performance of male controls compared to female controls. This contrasts with the prevalence of Alzheimer's disease in humans, which is higher in women than in men (Mazure and Swendsen, 2016). Women diagnosed with Alzheimer's disease also show a faster progression of hippocampal atrophy than men (Ardekani et al., 2016). The underlying causes of these differences are unclear, although it is known that women who are positive for the ε4 allele of the apolipoprotein E gene are at greater risk of developing Alzheimer's than are men with this allele (Riedel et al., 2016). Interestingly, most studies of 5XFAD mice have reported no sex differences (Oakley et al., 2006; O'Leary et al., 2017), although female 5XFAD mice do show accelerated hippocampal β-amyloidosis in response to stress, compared to males (Devi et al., 2010). Future work aimed at understanding the mechanistic basis for the sex differences in gap detection in 5XFAD mice could help shed light on the basis of sex differences in Alzheimer's disease in humans.

We found that gap detection performance developed with age in control mice, improving steeply before reaching stable adult performance at about 60 days of age. 5XFAD mice showed a similar developmental trajectory. This is the first characterization of the development of gap detection in juvenile mice, although similar results have been reported for rats, which show improvement of gap detection thresholds up until reaching an adult level at 35 days of age (Dean et al., 1990; Friedman et al., 2004). Humans show a similar developmental improvement of gap detection thresholds from infancy through childhood to adulthood (Werner et al., 1992; Trehub et al., 1995; Smith et al., 2006).

How is information processing in neural circuits impaired in 5XFAD mice? It is possible that gap detection deficits could arise from either cortical or subcortical pathology, or both. Gap detection recruits multiple levels of the auditory system, from the brainstem and midbrain that mediate startle reflexes and pre-pulse inhibition (Parham and Willott, 1990; Koch, 1999), to thalamus and auditory cortex that are critically involved in the detection of brief gaps (< 64 ms) but not longer gaps (Weible et al., 2014a,b). 5XFAD mice show much more severe pathology in cortex than in thalamus or brainstem (Hall and Roberson, 2012). These mice show rapid accumulation of Aβ42 in the brain and robust plaque deposition starting at 1.5–2 months of age, first appearing in deep layers of cortex and the subiculum, and then spreading to fill much of cortex, subiculum, and hippocampus. Fewer plaques are seen in the brainstem and thalamus. Neuronal loss starting between 4 and 9 months is marked by loss of large layer 5 pyramidal cells (Hall and Roberson, 2012), which project from auditory cortex to inferior colliculus and are thought to mediate cortical involvement in brief gap detection. The specificity for layer 5 pyramidal cells may arise from their strong expression of the *Thy1* gene, which is the promoter that drives 5XFAD transgene expression (Oakley et al., 2006).

The fact that 5XFAD mice show detection deficits for both brief and long gaps raises the possibility that the underlying circuit damage is not just cortical but also includes subcortical regions such as auditory brainstem or midbrain. Indeed, these mice show elevated auditory brainstem response thresholds at 13–14 months, and inner and outer hair cell loss at 15–16 months (O'Leary et al., 2017). It remains a key open question whether the gap detection deficits we observed starting at 60 days could arise in part from early precursors of such peripheral damage, or whether they instead arise from early degeneration of cortical circuits or the cortico-collicular pathway. This question is highly relevant to human Alzheimer's etiology, because it remains unclear which aspect of neuropathology underlies human gap detection and speech encoding deficits. On one hand, there is evidence for subcortical deficits in speech processing in MCI patients (Bidelman et al., 2017), while on the other hand there is evidence that cortical thinning and reduced hippocampal/entorhinal volumes are correlated with gap detection deficits in these patients (Iliadou et al., 2017). Mouse models of Alzheimer's disease represent an exciting opportunity to elucidate how neural circuit alterations underlie gap detection deficits. For example, neurophysiological studies can assess whether and how gap encoding is altered at key levels of the auditory hierarchy, from auditory brainstem, to inferior colliculus, to auditory thalamus and cortex. In particular, encoding of sound offsets is known to be altered at the level of auditory thalamus in a mouse model of neurodevelopmental disorders, which appears to account for gap detection deficits in these mice (Anderson and Linden, 2016). Similar mechanisms could contribute to gap detection deficits in 5XFAD mice. Understanding how specific alterations in gap detection circuitry underlie behavioral gap detection deficits will be a vital step in validating this measure as a biomarker for early Alzheimer's diagnosis.

## Methods

### Mice

We used heterozygous 5XFAD mice (*n* = 27 mice, stock number 006554, The Jackson Laboratory) on a C57BL6/SJ1 hybrid background (stock number 100012, The Jackson Laboratory), with wildtype littermates as controls (*n* = 25 mice). This background is heterozygous for the retinal degeneration mutation *Pde6b*^*rd*1^, which causes blindness in *Pde6b*^*rd*1^ homozygous mice; we excluded offspring that were *Pde6b*^*rd*1^ homozygous.

### Behavior

All behavioral data were collected in a sound-attenuating chamber. Sounds were delivered from a free-field speaker directly facing the animal. The speaker was calibrated to within ±1 dB using a Brüel and Kjær 4939 $\frac{1}{4}$″ microphone positioned where the ear would be, without the animal present. Mice were loosely restrained in a plastic tube (35 mm inner diameter, 1.5 mm wall thickness) affixed to a flat base (see Figure 1A). The tube was perforated (~3 mm diameter) to allow effective transmission of sound, with no more than 5 dB attenuation. To measure the startle response, the tube rested on a piezo transducer.

We measured gap detection using a variant of pre-pulse inhibition of the acoustic startle response, in which a gap that precedes a startle stimulus acts as a cue that reduces the magnitude of the startle response. Acoustic stimuli were embedded in continuous background white noise (80 dB SPL). Startle stimuli (25 ms white noise bursts, 100 dB SPL) were separated by a random intertrial interval of 20 ± 7 s. Silent gaps in the continuous background noise preceded the startle stimulus, separated by a 50 ms interval between the end of the gap and the onset of the startle stimulus. Gap durations were 1, 2, 3, 4, 8, 32, or 256 ms, with 20 presentations per session, and did not include ramps at onset or offset. We also presented “pure startle” stimuli in isolation, without a gap.

### Data Analysis

We quantified startle responses by calculating the peak of the rectified startle response signal in a 100 ms window following startle stimulus onset. We quantified gap detection as the percentage reduction in the median startle response compared to the median pure startle response for each mouse. Because this measure depends on the startle response, we verified that mice exhibited robust startle responses by comparing the median pure startle response to an equivalent pre-stimulus baseline period (*t*-test with criterion *p* < 0.001), resulting in the exclusion of eight sessions in 5XFAD mice and six session in control mice (due to a mixture of reduced startle magnitudes and increased baseline activity). In some cases, startle responses were slightly facilitated following a gap (e.g., for 1 ms gaps), which results in a negative gap detection value (i.e., a negative reduction in startle).

To test for group differences between 5XFAD and control mice, we used the Kruskal-Wallis test (non-parametric alternative to the 1-way ANOVA) for main effects across sessions, and then used the Wilcoxon rank-sum *post-hoc* to test for effects at individual gap durations.

Mice were tested approximately once per week from ages 25 to 136 days, although not all mice were tested at all age points (average: 4.2 sessions per mouse, range: 1–14). To determine at which age the deterioration in 5XFAD mice was first detectable, we used a 1-tailed rank-sum test on 256 ms gap detection in age bins increasing in 10 day increments (e.g., 25–40 days, 25–50 days, etc.). This test showed no difference earlier than 25–60 days and a significant difference for all bins 25–60 days and longer, from which we conclude that the deficit in 5XFAD mice is first detectable at 60 days. We obtained the identical result (the deficit was first detectable at 60 days) when we repeated this analysis testing only 10-day bins (e.g., 30–40 days, 40–50 days, etc.).

To model the effects of age (Figures 3C,D), which showed both a developmental improvement in young mice and a degenerative decline in older mice, we fit gap detection performance using a two-exponential fit of the form

$$\begin{array}{l} y=A\cdot \left(e^{\frac{-\left(t-t_{\text{o}}\right)}{\tau_{1}}}-e^{\frac{-\left(t-t_{\text{o}}\right)}{\tau_{2}}}\right) \end{array}$$

where A corresponds to a maximal level of gap detection, *t*_o_ corresponds to the developmental onset of gap detection, and τ_1_ and τ_2_ are time constants governing the developmental increase and age-related decline of gap detection. To test for the significance of the developmental improvement in young mice and the degenerative decline in older mice, we used linear regression to fit gap detection as a function of age separately for ages 25–60 days and 60–136 days (Table 1).

To jointly model gap detection as a function of genotype, gap duration, age, and sex, we used a generalized piecewise linear mixed effects model (Table 2). This model accounts for the random effects of individual mice, modeled as intercepts and slopes as a function of gap duration and age. This allows the model to account for the fact that not all mice were tested at all age points. We fit age with a piecewise linear function split before and after 60 days (denoted as “age_early” and “age_late”). We used the full model given by:

gap detection ~ genotype + gap duration + age_early + age_late + sex
+ genotype:gap duration + genotype:age_early + genotype:age_late
+ genotype:sex + gap duration:age_early + gap duration:age_late
+ gap duration:sex + age_early:sex + age_late:sex
+ (1 + gap duration | mouse) + (1 + age_early | mouse)
+ (1 + age_late | mouse)

where “~” denotes “modeled as,” “:” denotes interaction products, “|” denotes “by,” which accounts for the random effects of individual mice modeled as random intercepts (“1”) and random slopes for the dependence on gap duration and age. We tested for the significance of model predictors using ANOVA.

## Data Availability

Data from this study are available upon request.

## Ethics Statement

All procedures were in accordance with the National Institutes of Health guidelines, as approved by the University of Oregon Institutional Animal Care and Use Committee.

## Author Contributions

KK, AS, and AW performed the experiments. MW designed the experiments, analyzed the data, and wrote the paper.

### Conflict of Interest Statement

The authors declare that the research was conducted in the absence of any commercial or financial relationships that could be construed as a potential conflict of interest.
